# Racial disparities in pancreatic neuroendocrine tumors survival: a SEER study

**DOI:** 10.1002/cam4.1220

**Published:** 2017-10-04

**Authors:** Huaqiang Zhou, Yuanzhe Zhang, Xiaoyue Wei, Kaibin Yang, Wulin Tan, Zeting Qiu, Si Li, Qinchang Chen, Yiyan Song, Shaowei Gao

**Affiliations:** ^1^ Department of Anesthesiology The First Affiliated Hospital of Sun Yat‐sen University Guangzhou China; ^2^ Zhongshan School of Medicine Sun Yat‐sen University Guangzhou China

**Keywords:** Pancreatic neuroendocrine tumors, prognosis, racial disparity, SEER, survival analysis

## Abstract

Pancreatic neuroendocrine tumor (pancreatic NETs), is an important cause of cancer‐related death worldwide. No study has rigorously explored the impact of ethnicity on pancreatic NETs. We aimed to demonstrate the relationship between ethnicity and the survival of patients with pancreatic NETs. We used the SEER database to identify patients with pancreatic NETs from 2004 to 2013. Kaplan–Meier methods and Cox proportional hazard models were used to evaluate the impact of race on survival in pancreatic NETs patients. A total of 3850 patients were included: 3357 Non‐Blacks, 493 Blacks. We stratified races as “Black” and “White/Other.” Blacks were more likely to be diagnosed with later stages of tumors (*P *=* *0.021). As for the treatment, the access to surgery seemed to be more limited in Blacks than non‐Black patients (*P *=* *0.012). Compared with non‐Black patients, Black patients have worse overall survival (OS) (HR = 1.17, 95% CI: 1.00–1.37, *P *=* *0.046) and pancreatic neuroendocrine tumors specific survival (PNSS) (HR = 1.22, 95% CI: 1.01–1.48, *P *=* *0.044). Multivariate Cox analysis identified that disease extension at the time of diagnosis and surgical status contributed to the ethnical survival disparity. Black patients whose stages at diagnosis were localized had significantly worse OS (HR = 2.09, 95% CI: 1.18–3.71, *P *=* *0.011) and PNSS (HR = 3.79, 95% CI: 1.62–8.82, *P *=* *0.002). As for the patients who did not receive surgery, Blacks also have a worse OS (HR = 1.18, 95% CI: 1.00–1.41, *P *=* *0.045). The Black patients had both worse OS and PNSS compared to non‐Black patients. The restricted utilization of surgery, and the advanced disease extension at the time of diagnosis are the possible contributors to poorer survival of Blacks with pancreatic NETs.

## Introduction

Pancreatic neuroendocrine tumors (pancreatic NETs) are rare pancreatic neoplasms with an annual incidence of 1 per 100,000 individuals. The incidence has been rising in the United States and elsewhere over the last two decades, which is due to recent improvements in detecting pancreatic NETs 1, 2, 3. The tumors are categorized as functional or nonfunctional, and approximately 10–30% of pancreatic NETs are functional 4. They account for 3–5% of pancreatic malignancies and overall have a better prognosis than pancreatic exocrine tumors 5. The overall 5‐year relative survival rate of pancreatic NETs is approximately 42% 5. There are some prognostic factors of pancreatic NETs being reported in studies, including tumor‐size, histologic grade, TNM stage, treatment strategy, and marital status 6, 7. Studies about the impact of race and ethnicity on tumors have published widely in recent years 8, 9, 10. Black patients or African American patients are associated with the poor overall survival in a variety of tumors. However, no study has rigorously explored the impact of ethnic disparity on survival of patients with pancreatic NETs. Our study aimed to demonstrate the relationship between ethnic disparity and survival in pancreatic NETs patients using the Surveillance, Epidemiology, and End Results (SEER) database.

## Materials and Methods

### Patients selection

The SEER*Stat software version 8.3.2 (accession number: 13693‐Nov2015) was used to extract data from the SEER database 11, 12. We identified patients diagnosed with pancreatic NETs that were reported to the SEER database from 2004 to 2013. ICD‐O‐3 (International Classification of Diseases for Oncology, 3rd edition) morphology codes 8150, 8151, 8152, 8153, 8155, 8156, 8157, 8240, 8241, 8242, 8243, 8246, and 8249 were used to identify pancreatic NETs. All pancreatic anatomical sites (C25.0—C25.9) were included in the study 13.

The exclusion criteria were as follows: (1) who had more than one primary cancer and the pancreatic NETs was not the first; (2) incomplete follow‐up information or unknown survival length; (3) and who had an unknown cause of death; (4) and who had incomplete county‐level socioeconomic data 14.

### Variable definition

We included variabilities as sex, age, marital status, education, income, tumor location, tumor size, metastatic status, histologic type, pathology grade, extent of disease, TNM stage, surgery, and race. We grouped races as “Black” and “White/Other” (non‐Black). Marital status was divided as married, unmarried, and unknown. We included socioeconomic status (SES, an economic, and sociological combined total measure) related variables as following: education (the percentage of adults aged ≥25 years who <12 years of education), poverty (the percentage of individuals living below the poverty line), and income (median annual household income). These variables were used as continuous variables in this study. According to the definitions of Country Attributes in SEER data, the higher values of the variables of education and poverty are, the lower the values of SES are. SEER staging was used to define disease extension: localized, regional and distant 15. CS Mets at Dx(metastatic status) identifies whether there is metastatic involvement of distant site(s) at the time of diagnosis. CS Mets at Dx is part of the Collaborative Stage Data Collection System (CS), and was first introduced in 2004. It is used to derive some American Joint Committee on Cancer TNM Staging System (AJCC) M values and SEER Summary Stage codes 16. The CS Mets at Dx was introduced into yes, no and unknown. We set 2 and 4 cm as the cutoff points of tumor size according to The European Neuroendocrine Tumor Society Staging Classification (ENETS) 17.

### Outcomes

The primary outcomes of this study were overall survival and pancreatic neuroendocrine tumors specific survival. OS was defined as the time from diagnosis to date of any death. pancreatic neuroendocrine tumors specific survival (PNSS) was derived from the time of diagnosis to date of pancreatic NETs cancer‐specific death. Death attributed to pancreatic NETs was regarded as an event. Patients who died from other causes or were still alive at the follow‐up cutoff date were treated as censored observations 14. The follow‐up cutoff date was December 31, 2013.

### Statistical analyses

The baseline patients’ demographic characteristics, tumor characteristics, and treatments were compared by Mann–Whitney *U* test (continuous variables) or Pearson chi‐squared test (categorical variables). Kaplan–Meier curve was used to describe the overall survival, pancreatic neuroendocrine tumors specific survival and the differences between groups were tested by log‐rank method. Factors significantly relating to outcomes in the univariate analysis were selected for the Cox proportional hazard model for recognizing confounding factors. Thus, we wound see whether race impacted the survival time of pancreatic NETs. We also stratified each ethnic group into different SEER stages at diagnosis and different surgical status, so that subgroup analysis was carried out. Statistical significance was set at two‐sided *P *<* *0.05. All statistical analyses were performed using R 3.3.2 (R Foundation for Statistical Computing, Vienna, Austria; www.r-project.org).

## Results

### Population characteristics

Based on selection criteria, this retrospective cohort study included a total of 3852 patients who were diagnosed with pancreatic NETs from 2004 to 2013. The population characteristics are summarized in Table 1. Among them, 3357 (87%) were non‐Black patients, 493 (13%) were Blacks (*P *<* *0.001). The Black patients had a lower percentage of males, younger average age and a lower marriage rate (*P *<* *0.001). The socioeconomic status of Blacks was significantly unfavorable than non‐Black patients’ condition, including education (*P *=* *0.051), higher poverty rate (*P *<* *0.001) and lower annual income (*P *<* *0.001). Blacks were more likely to be diagnosed with later stages of tumors (*P *=* *0.021). As for the treatment, the access to surgery seemed to be more limited in Blacks than non‐Black patients (*P *=* *0.012). We found no statistically significant differences between the ethnicities in other characteristics including tumor size, histology type, pathology grade and AJCC groups (*P *>* *0.05).

**Table 1:** Baseline demographic and tumor characteristics of patients in Surveillance, Epidemiology, and End Results (SEER) database

Characteristics	Total	White/Other	Black	*P* value
	3850 (100)	3357 (87.19)	493 (12.80)	
Sex				<0.001
Male	2118 (55.0)	1894 (56.4)	224 (45.4)	
Female	1732 (45.0)	1463 (43.6)	269 (54.6)	
Age at diagnosis (mean [SD])	59.36 (13.98)	59.78 (13.96)	56.54 (13.82)	<0.001
Marital status				<0.001
Married	2402 (62.4)	2201 (65.6)	201 (40.8)	
Unknown	181 (4.7)	153 (4.6)	28 (5.7)	
Unmarried	1267 (32.9)	1003 (29.9)	264 (53.5)	
Education (mean [SD])	14.98 (6.16)	14.91 (6.29)	15.49 (5.18)	0.051
Poverty (mean [SD])	14.23 (5.13)	13.90 (5.03)	16.50 (5.24)	<0.001
Income (mean (SD))^a^	6078.42 (1479.09)	6161.98 (1489.22)	5509.43 (1270.79)	<0.001
Primary Site				<0.001
Body	478 (12.4)	412 (12.3)	66 (13.4)	
Head	1180 (30.6)	1012 (30.1)	168 (34.1)	
Others	1046 (27.2)	894 (26.6)	152 (30.8)	
Tail	1146 (29.8)	1039 (31.0)	107 (21.7)	
Metastatic status				0.322
No	1975 (51.3)	1733 (51.6)	242 (49.1)	
Unknown	101 (2.6)	91 (2.7)	10 (2.0)	
Yes	1774 (46.1)	1533 (45.7)	241 (48.9)	
Tumor size				0.111
<2 cm	674 (17.5)	599 (17.8)	75 (15.2)	
>4 cm	1359 (35.3)	1175 (35.0)	184 (37.3)	
2–4 cm	1210 (31.4)	1067 (31.8)	143 (29.0)	
Unknown	607 (15.8)	516 (15.4)	91 (18.5)	
Histology				0.108
Functional	100 (2.6)	93 (2.8)	7 (1.4)	
Nonfunctional	3750 (97.4)	3264 (97.2)	486 (98.6)	
Grade				0.179
Grade I	1435 (37.3)	1265 (37.7)	170 (34.5)	
Grade II	371 (9.6)	325 (9.7)	46 (9.3)	
Grade III	258 (6.7)	219 (6.5)	39 (7.9)	
Grade IV	67 (1.7)	63 (1.9)	4 (0.8)	
Unknown	1719 (44.6)	1485 (44.2)	234 (47.5)	
Disease extension (%)				0.021
Distant	1875 (48.7)	1623 (48.3)	252 (51.1)	
Localized	1102 (28.6)	988 (29.4)	114 (23.1)	
Regional	780 (20.3)	669 (19.9)	111 (22.5)	
Unknown	93 (2.4)	77 (2.3)	16 (3.2)	
AJCC groups (%)				0.232
I/II	1144 (29.7)	1009 (30.1)	135 (27.4)	
III/IV	1562 (40.6)	1345 (40.1)	217 (44.0)	
Unknown	1144 (29.7)	1003 (29.9)	141 (28.6)	
Surgery (%)				0.012
Yes	1887 (49.0)	1676 (49.9)	211 (42.8)	
No	1951 (50.7)	1671 (49.8)	280 (56.8)	
Unknown	12 (0.3)	10 (0.3)	2 (0.4)	

a Income is displayed as dollars in tens in SEER database (e.g., 6688 represents $66,880).

### Ethnic disparity in OS in overall pancreatic NETs population

**Figure 1:**
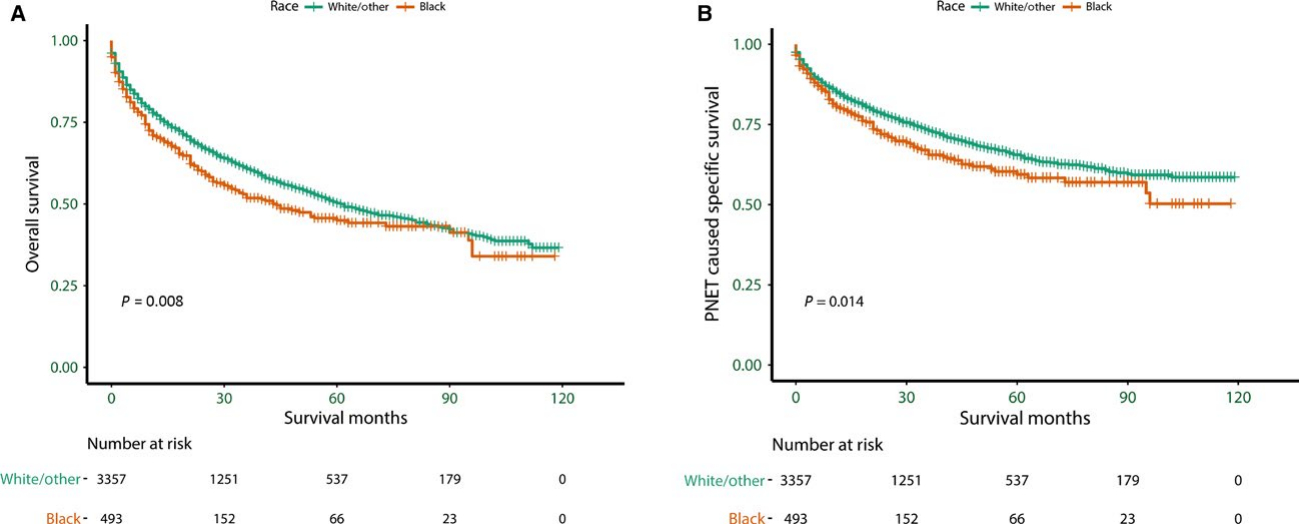
Survival curves in patients with pancreatic neuroendocrine tumors between Black and White/Other. (A) Overall survival (OS): *χ* ^2^ = 7.21, *P *=* *0.008. (B) Pancreatic neuroendocrine tumors cause specific survival (PNSS): *χ* ^2^ = 6.11, *P *=* *0.014.

As the OS Kaplan–Meier curve shown in Figure 1A, there was a significant survival difference according to race (log rank test *P *=* *0.008). Compared with other racial groups, Black patients have worse OS. The 5‐year OS was 45.0% in Black patients, 50.3% in other racial groups. Similarly, the median OS of Black patients (43 months) was lower than the others (61 months). According to univariate log‐rank test, several variables were closely associated with OS, including sex, age, marital status, education, poverty, income, primary site, tumor size, metastatic status, histologic type, pathology grade, extension of disease, AJCC stage, and surgical status. Race was still an independent prognostic factor after adjusting for aforementioned variables in the Cox proportional hazard regression model, and Black patients have worse OS than non‐Black patients (hazard ratio [HR] = 1.17, confidence interval [95% CI]: 1.00–1.37, *P *=* *0.046) (Table 2).

**Table 2:** Univariate and Multivariate survival analysis of OS in pancreatic endocrine tumor patients. Surveillance, Epidemiology, and End Results 2004–2013 (*n* = 3850)

Characteristic	Univariate analysis		Multivariate analysis		
	Log‐rank *χ* ^2^	*P* value	HR	95% CI	*P* value
Sex	8.77	0.003			
Female			Reference		
Male			1.16	1.04–1.29	0.008
Age at diagnosis	230.42	<0.001	1.03	1.02–1.03	<0.001
Marital status	38.52	<0.001			
Married			Reference		
Unknown			0.92	0.70–1.21	0.540
Unmarried			1.28	1.15–1.44	<0.001
Education	1.00	0.318	1.00	0.99–1.01	0.659
Poverty	5.87	0.015	1.01	0.99–1.03	0.502
Income	8.05	0.005	1.00	0.99–1.00	0.817
Primary site	58.06	<0.001			
Body			Reference		
Head			1.02	0.85–1.23	0.806
Tail			0.95	0.79–1.15	0.627
Others			1.07	0.89–1.29	0.485
Metastatic status	825.83	<0.001			
No			Reference		
Unknown			1.50	1.03–2.19	0.035
Yes			1.19	0.88–1.60	0.258
Tumor size	356.80	<0.001			
<2 cm			Reference		
>4 cm			1.25	0.98–1.60	0.074
2–4 cm			1.33	1.04–1.69	0.025
Unknown			1.42	1.10–1.84	0.007
Histology	14.06	<0.001			
Functional			Reference		
Nonfunctional			1.18	0.79–1.78	0.415
Grade	681.25	<0.001			
Grade II			Reference		
Grade I			0.85	0.67–1.08	0.174
Grade III			3.07	2.39–3.94	0.000
Grade IV			3.68	2.60–5.23	0.000
Unknown			1.41	1.14–1.75	0.001
Disease extension	828.12	<0.001			
Distant			Reference		
Localized			0.41	0.29–0.6	<0.001
Regional			0.83	0.60–1.16	0.277
Unknown			0.55	0.34–0.89	0.016
AJCC groups	783.26	<0.001			
I/II			Reference		
III/IV			1.55	1.20–2.00	0.001
Unknown			1.10	0.87–1.39	0.430
Surgery	1041.55	<0.001			
No			Reference		
Yes			0.38	0.32–0.45	<0.001
Unknown			1.53	0.75–3.09	0.241
Race	7.21	0.008			
White/Other			Reference		
Black			1.17	1.00–1.37	0.046

### Ethnic disparity in PNSS in overall pancreatic NETs population

As the PNSS Kaplan–Meier curve shown in Figure 1B, Blacks have worse PNSS compared to non‐Black patients (*P *=* *0.014). The 5‐year PNSS of Blacks is 59.4%, and 65.2% in other racial groups. The median PNSS of Black patients (96 months) is still lower than non‐Black patients (over 103 months). Univariate log‐rank test shows that several variables are closely associated with PNSS, including sex, age, marital status, education, poverty, income, primary site, tumor size, metastatic status, histologic type, pathology grade, extent of disease, AJCC stage, and surgical status. The multivariate Cox regression analysis show that race is still an independent prognostic factor, and Black patients have worse PNSS than non‐Black patients (HR = 1.22, 95% CI: 1.01–1.48, *P *=* *0.044) (Table 3).

**Table 3:** Univariate and Multivariate survival analysis of PNSS in pancreatic endocrine tumor patients. Surveillance, Epidemiology, and End Results 2004–2013 (*n* = 3850)

Characteristic	Univariate analysis		Multivariate analysis		
	Log‐rank *χ* ^2^	*P* value	HR	95% CI	*P* value
Sex	9.86	0.002			
Female			Reference		
Male			1.18	1.03–1.35	0.020
Age at diagnosis	144.28	<0.001	1.03	1.02–1.03	<0.001
Marital status	16.50	<0.001			
Married			Reference		
Unknown			0.88	0.62–1.26	0.490
Unmarried			1.20	1.04–1.38	0.014
Education	1.74	0.187	1.01	0.99–1.02	0.291
Poverty	4.07	0.044	1.00	0.97–1.02	0.744
Income	6.78	0.009	1.00	0.99–1.00	0.360
Primary site	43.48	<0.001			
Body			Reference		
Head			1.22	0.97–1.55	0.093
Tail			1.00	0.78–1.29	0.974
Others			1.12	0.87–1.43	0.379
Metastatic status	563.69	<0.001			
No			Reference		
Unknown			1.81	1.13–2.90	0.014
Yes			1.12	0.79–1.60	0.529
Tumor size	179.55	<0.001			
<2 cm			Reference		
>4 cm			1.14	0.83–1.56	0.407
2–4 cm			1.21	0.88–1.66	0.239
Unknown			1.11	0.80–1.54	0.545
Histology	16.04	<0.001			
Functional			Reference		
Nonfunctional			1.73	0.91–3.29	0.096
Grade	553.52	<0.001			
Grade II			Reference		
Grade I			0.77	0.56–1.05	0.101
Grade III			3.47	2.55–4.73	<0.001
Grade IV			3.97	2.61–6.06	<0.001
Unknown			1.45	1.10–1.90	0.007
Disease extension	587.99	<0.001			
Distant			Reference		
Localized			0.24	0.15–0.39	<0.001
Regional			0.82	0.55–1.22	0.336
Unknown			0.41	0.22–0.75	0.004
AJCC groups	558.94	<0.001			
I/II			Reference		
III/IV			1.64	1.17–2.29	0.004
Unknown			1.16	0.86–1.59	0.333
Surgery	682.66	<0.001			
No			Reference		
Yes			0.37	0.30–0.45	<0.001
Unknown			1.16	0.43–3.13	0.772
Race	6.11	0.014			
White/Other			Reference		
Black			1.22	1.01–1.48	0.044

### Ethnic disparity in OS and PNSS in patients stratified by SEER stage

We also explored the survival patterns among ethnicities in subgroups stratified by SEER stage at diagnosis: localized (28.6%), regional (20.2%), and distant (48.7%) (*P *=* *0.003). We excluded the unstaged patients and patients whose stages at diagnosis were unknown, which only added up to 2.4% of the total. The patients whose stages at diagnosis were localized accounted for 28.6% of the total patients: 23.1% of Blacks (*n* = 114), 29.4% of non‐Blacks (*n* = 988). The patients whose stages at diagnosis were regional accounted for 20.2% of the total patients: 22.5% of Blacks (*n* = 111), 19.9% of non‐Blacks (*n* = 669). The patients whose stages at diagnosis were distant accounted for 48.7% of the total patients: 51.1% of Blacks (*n* = 252), 48.3% of non‐Blacks (*n* = 1623).

**Figure 2:**
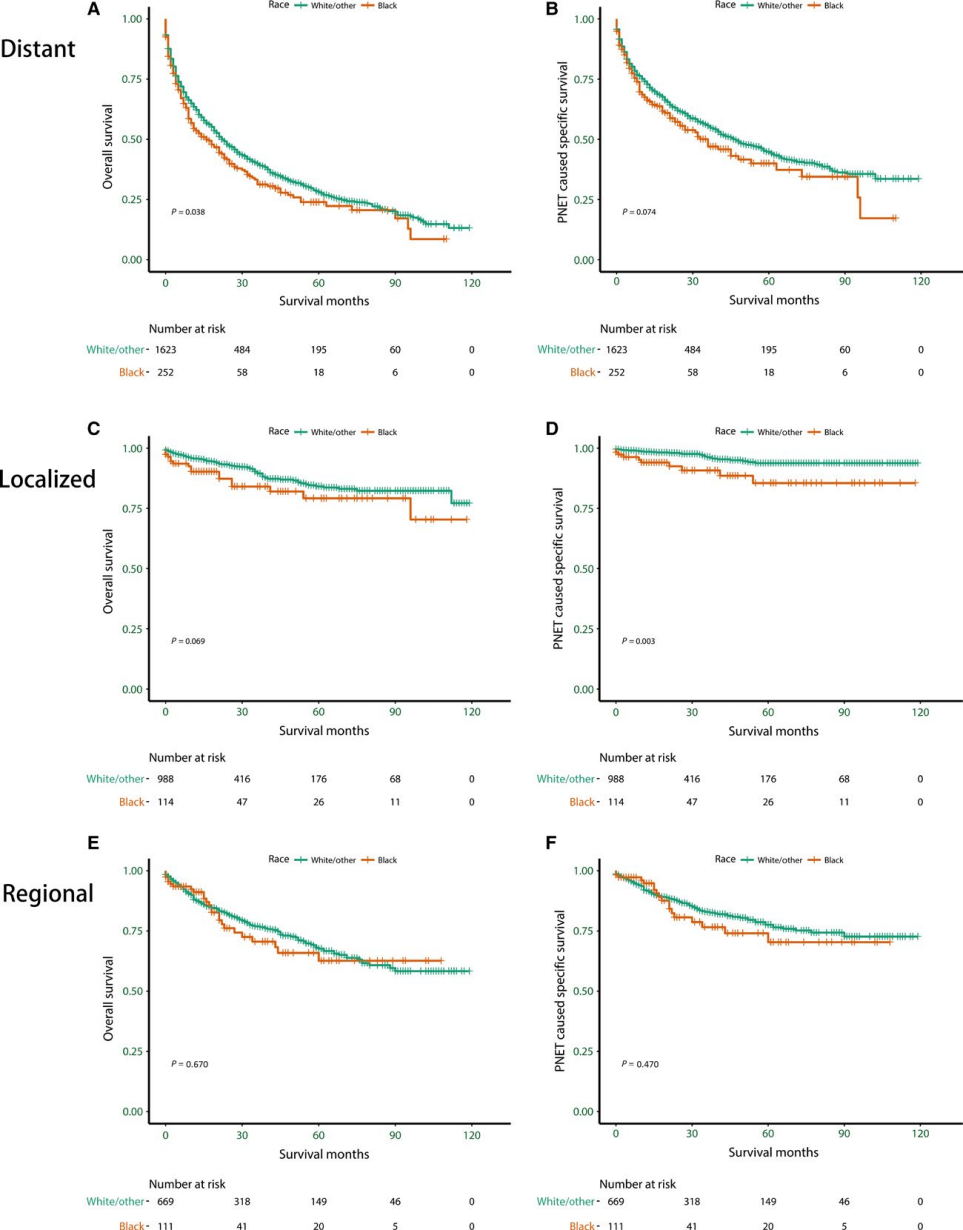
Survival curves in different Surveillance, Epidemiology, and End Results (SEER) stage subgroup patients with pancreatic neuroendocrine tumors according to race. (A). Distant, Overall survival (OS): *χ* ^2^ = 4.32, *P *=* *0.037; (B). Distant, Pancreatic neuroendocrine tumors cause specific survival (PNSS): *χ* ^2^ = 3.19, *P *=* *0.073; (C). Localized, OS:* χ* ^2^ = 3.31, *P *=* *0.068; (D). Localized, PNSS:* χ* ^2^ = 8.76, *P *=* *0.003; (E). Regional, OS:* χ* ^2^ = 0.19, *P* = 0.666; (F). Regional, PNSS:* χ* ^2^ = 0.53, *P *=* *0.465.

As is shown in Figure 2C, for the patients whose stages at diagnosis were localized, of interest, Blacks (HR = 2.09, 95% CI: 1.18–3.71, *P *=* *0.011) had an extremely worse OS compared to non‐Black patients. And the PNSS for Blacks (HR = 3.79, 95% CI: 1.62–8.82, *P *=* *0.002), shown in Figure 2D, was extraordinarily worse compared to non‐Black patients. As for the patients whose stages at diagnosis were regional and distant, the survival disparity of Blacks and non‐Blacks was not significant (Fig. 2 A, B, E and F, Table 4).

**Table 4:** Univariate and Multivariate survival analysis of pancreatic endocrine tumor survival based on different extension. Surveillance, Epidemiology, and End Results 2004–2013 (*n* = 3850)

Characteristic	Univariate analysis		Multivariate analysis		
	Log‐rank *χ* ^2^	*P* value	HR	95% CI	*P* value
Overall survival					
*Localized*	3.31	0.068			
White/Other			Reference		
Black			2.09	1.18–3.71	0.011
*Distant*	4.32	0.037			
White/Other			Reference		
Black			1.17	0.98–1.39	0.086
*Regional*	0.19	0.666			
White/Other			Reference		
Black			1.06	0.68–1.66	0.797
PNET cause‐specific survival					
*Localized*	8.76	0.003			
White/Other			Reference		
Black			3.79	1.62–8.82	0.002
*Distant*	3.19	0.073			
White/Other			Reference		
Black			1.22	0.98–1.52	0.072
*Regional*	0.53	0.465			
White/Other			Reference		
Black			1.16	0.68–1.98	0.584

PNET, pancreatic neuroendocrine tumor (pancreatic NETs).

### Ethnic disparity in OS and PNSS in patients stratified by surgical status

**Figure 3:**
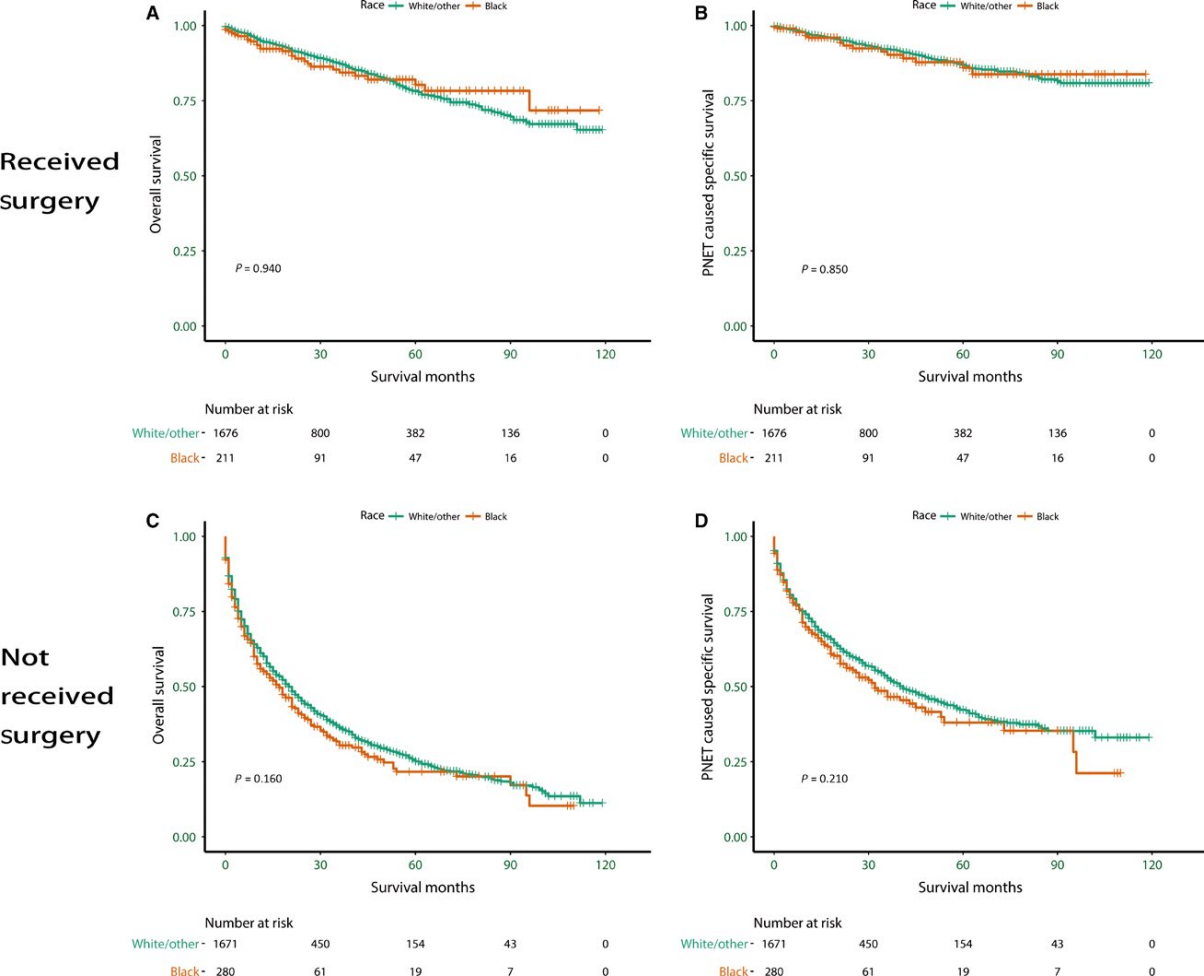
Survival curves in different surgical status subgroup patients with pancreatic neuroendocrine tumors according to race. (A). Received Surgery, Overall survival (OS): *χ* ^2^ = 0.01, *P *=* *0.937; (B). Received Surgery, Pancreatic neuroendocrine tumors cause specific survival (PNSS): *χ* ^2^ = 0.04, *P *=* *0.850; (C). Not Received Surgery, OS:* χ* ^2^ = 1.94, *P *=* *0.164; (D). Not Received Surgery, PNSS:* χ* ^2^ = 1.56, *P *=* *0.212.

We also explored the survival patterns among ethnicities in subgroups stratified by surgical status: surgery performed (50.7%), and no surgery performed (49.0%) (*P *=* *0.012). We excluded the patients with unknown surgical status (0.3%). The patients who received surgery accounted for 50.7% of the total, 42.8% of Blacks (*n* = 211), and 49.9% of non‐Blacks (*n* = 1676). The patients who did not receive surgery accounted for 49.0% of the total, 56.8% of Blacks (*n* = 280), and 49.8% of non‐Blacks (*n* = 1671). As for the patients who did not receive surgery, Blacks (HR = 1.18, 95% CI: 1.00–1.41, *P *=* *0.045) have a significantly worse OS than non‐Black patients (Fig. 3C). The PNSS for Blacks (HR = 1.21, 95% CI: 0.98–1.50, *P *=* *0.071), however, has no significant disparity (Fig. 3D). For the patients who received surgery, both the OS (HR = 1.07, 95% CI: 0.71–1.61, *P *=* *0.74) and PNSS (HR = 1.20, 95% CI: 0.71–2.02, *P *=* *0.50) for Blacks display no significant disparity (Fig. 3A, B, Table 5).

**Table 5:** Univariate and Multivariate survival analysis of pancreatic endocrine tumor survival based on surgical status. Surveillance, Epidemiology, and End Results 2004–2013 (*n* = 3850)

Characteristic	Univariate analysis		Multivariate analysis		
	Log‐rank *χ* ^2^	*P* value	HR	95% CI	*P* value
Overall survival					
*Received surgery*	0.01	0.937			
White/Other			Reference		
Black			1.07	0.71–1.61	0.744
*Not received surgery*	1.94	0.164			
White/Other			Reference		
Black			1.19	1.00–1.41	0.045
PNET cause‐specific survival					
*Received surgery*	0.04	0.850			
White/Other			Reference		
Black			1.20	0.71–2.02	0.496
*Not received surgery*	1.56	0.212			
White/Other			Reference		
Black			1.21	0.98–1.50	0.071

PNET, pancreatic neuroendocrine tumor (pancreatic NETs).

## Discussion

Our study shows the racial disparities among pancreatic NETs patients. As shown in the univariable and multivariable analysis, the Black patients have worse OS and PNSS than other racial groups, even after adjusting for sex, age, marital status, presence of distant metastasis, tumor size, histology, grade, AJCC stage, disease extension, and therapies. Actually, Yao and Dasari have reported the same worse overall survival for African American patients with pancreatic NETs, but without in‐depth exploration about factors influencing outcome 18, 19. We want to explore the reasons behind the phenomenon to have a better understanding of its contributors, which will provide useful inventions to reduce the disparity. Several factors have previously been studied in articles about ethnic disparity in survival for other tumors, such as marital status, SES, advanced stage and treatment 8, 9, 10.

According to the demographics of SEER patients, Blacks had a more advanced tumor stage at the moment of diagnosis comparing to non‐Black patients. Several studies supported the reason for delayed diagnosis may be due to Blacks’ low marriage rate and unfavorable SES 20, 21, 22. Previous studies demonstrated that marriage is associated with earlier stage at diagnosis and more favorable survival for various cancer type 22, 23, 24, 25, 26. It is also the case in pancreatic NETs. Our previous study revealed that being married contributes to a better survival in pancreatic NETs patients 14. Also, SES could be the potential contributor to ethnical survival disparity as it can affect the usage of medical resources of pancreatic NETs patients. Julien et al. found that SES are associated with treatment choice in pancreatic NETs 27. Blacks are more likely to be in disadvantaged SES, and tend to cluster in low quality hospital 28, 29.

Of interest, our subgroup analysis demonstrates that Blacks whose stage at diagnosis were localized had significantly worse OS and PNSS compared with non‐Black patients. However, we found no special disparity in distant and regional patients, that means race was an independent prognostic factor for OS and PNSS in localized disease, but not in regional disease or distant stage disease. We speculated that the reason may be that effective health care was not available to Blacks in the early stage.

Our analysis showed that access to effective options of treatment may be a reason for the poor survival of Blacks. The only curative treatment of pancreatic NETs is radical surgery. The majority of studies have demonstrated that Black patients with pancreatic cancer are less likely to undergo resection and have worse outcomes compared with white patients 8, 13, 16. Our study agreed with the above: Blacks had less surgical treatment compared to Whites and Others (42.8% vs. 49.9%, *P *=* *0.012). As for those patients underwent surgery, the OS and PNSS display no significant racial disparity. However, contrary to our study, a pancreatic NETs research based on National Cancer Database reported that race was not associated with treatment choice or survival in pancreatic NETs 27. The possible reason may be different select criteria, and what they focused on were patients with non‐metastatic. Our results suggested that surgery may be underused for Blacks due to unfavorable SES. And maximizing surgery rates appropriately may be good for reducing racial disparities for pancreatic NETs.

Though those factors above may partly contribute to the poor survival of Blacks, in our multivariate analysis, we could still observe the influence on pancreatic NETs’ survival by race itself. We made a hypothesis that genetic differences between races and ethnicities may be a significant reason to pancreatic NETs survival disparity. Previous studies on racial disparities in cancer attribute some of the difference in survival to tumor biology and genetic variation, especially in prostate cancer and breast cancer 30, 31, 32. For instance, a study showed that miR‐24 expression was linked to a racial difference between African‐American and Caucasian–American, which could contribute to race‐related tumorigenesis in prostate cancer 30. Also, for multiple myeloma, African American's higher levels of phenotypic heterogeneity and monoclonal immunoglobulin levels caused the unfavorable survival, compared with their white counterparts 33. Compared with Whites, African Americans have also been proved to have worse survival in other tumors due to racial genetic differences, including head and neck squamous cell carcinoma, kidney cancer 34, 35. However, no study has specifically explored pancreatic NETs‐related genetic differences in races and ethnicities and their contribution to pancreatic NETs survival disparity. Based on these findings above, we speculated that genetic differences may contribute to race disparity in pancreatic NETs survival. Further studies are required.

Our study also had limitations. First, as pancreatic NETs is a rare disease, the sample size is relatively small. Second, the SEER database does not provide patient‐level socioeconomic information, and the county‐level information can not reflect the socioeconomic condition of individual patients, which may have a significant impact on patients’ access to medical care and their survival. Third, the clinical details are not available from SEER database, which may affect the use of therapies and the survival. Fourth, the recurrence information is unavailable, so the differences in recurrence rates could not be examined. Finally, for lack of evidence, we were unable to know how ethnic disparity effected on survival due to our research design. Further studies are required to gain more evidence to confirm the findings.

## Conclusion

In conclusion, our study has found that Blacks had unfavorable OS and PNSS compared with non‐Black patients. Poor prognosis of Black patients may be associated with advanced disease extension at diagnosis, limited utilization of surgery. Also, we speculated that the ethnicity‐related genetic differences may contribute to the survival disparity of pancreatic NETs patients. Further studies are required on our findings.

## Conflicts of Interest

All of the authors have no conflicts of interest to declare.

## References

[1] Kloppel, G. , A. Perren , and P. U. Heitz . 2004 The gastroenteropancreatic neuroendocrine cell system and its tumors: the WHO classification. Ann. N. Y. Acad. Sci. 1014:13–27. 15153416 10.1196/annals.1294.002

[2] Klimstra, D. S. 2007 Nonductal neoplasms of the pancreas. Mod. Pathol. 20(Suppl 1):S94–S112. 17486055 10.1038/modpathol.3800686

[3] Hallet, J. , C. H. Law , M. Cukier , R. Saskin , N. Liu , and S. Singh . 2015 Exploring the rising incidence of neuroendocrine tumors: a population‐based analysis of epidemiology, metastatic presentation, and outcomes. Cancer 121:589–597. 25312765 10.1002/cncr.29099

[4] Liakakos, T. , and D. H. Roukos . 2011 Everolimus and sunitinib: from mouse models to treatment of pancreatic neuroendocrine tumors. Future Oncol. 7:1025–1029. 21919689 10.2217/fon.11.88

[5] Ries, L. A. G. , J. L. Young , G. E. Keel , M. P. Eisner , Y. D. Lin , and M. Horner , eds. 2007 SEER survival monograph: cancer survival among adults: U.S. SEER program, 1988–2001, patient and tumor characteristics. National Cancer Institute, SEER Program. NIH Pub. No. 076215.

[6] Han, X. , X. Xu , D. Jin , D. Wang , Y. Ji , and W. Lou . 2014 Clinicopathological characteristics and prognosis‐related factors of resectable pancreatic neuroendocrine tumors: a retrospective study of 104 cases in a single Chinese center. Pancreas 43:526–531. 24658317 10.1097/MPA.0000000000000065 PMC4206386

[7] Yang, M. , B. L. Tian , Y. Zhang , A. P. Su , P. J. Yue , S. Xu , et al. 2014 Evaluation of the World Health Organization 2010 grading system in surgical outcome and prognosis of pancreatic neuroendocrine tumors. Pancreas 43:1003–1008. 24945681 10.1097/MPA.0000000000000153

[8] Clegg, L. X. , F. P. Li , B. G. Hankey , K. Chu , and B. K. Edwards . 2002 Cancer survival among US whites and minorities ‐ A SEER (Surveillance, Epidemiology, and End Results) program population‐based study. Arch. Intern. Med. 162:1985–1993. 12230422 10.1001/archinte.162.17.1985

[9] Farmer, M. M. , and K. F. Ferraro . 2005 Are racial disparities in health conditional on socioeconomic status? Soc. Sci. Med. 60:191–204. 15482878 10.1016/j.socscimed.2004.04.026

[10] Mathur, A. K. , N. H. Osborne , R. J. Lynch , A. A. Ghaferi , J. B. Dimick , and C. J. Sonnenday . 2010 Racial/ethnic disparities in access to care and survival for patients with early‐stage hepatocellular carcinoma. Arch. Surg. 145:1158–1163. 21173289 10.1001/archsurg.2010.272

[11] Surveillance Research Program, National Cancer Institute SEER*Stat software (www.seer.cancer.gov/seerstat) version 8.3.2.

[12] Surveillance, Epidemiology, and End Results (SEER) Program (www.seer.cancer.gov) SEER*Stat Database: Incidence ‐ SEER 18 Regs Research Data + Hurricane Katrina Impacted Louisiana Cases, Nov 2015 Sub (1973‐2013 varying) ‐ Linked To County Attributes ‐ Total U.S., 1969–2014 Counties, National Cancer Institute, DCCPS, Surveillance Research Program, Surveillance Systems Branch, released April 2016, based on the November 2015 submission.

[13] Luo, G. , A. Javed , J. R. Strosberg , K. Jin , Y. Zhang , C. Liu , et al. 2017 Modified staging classification for pancreatic neuroendocrine tumors on the basis of the American Joint Committee on Cancer and European Neuroendocrine Tumor Society Systems. J. Clin. Oncol. 35:274–280. 27646952 10.1200/JCO.2016.67.8193

[14] Zhou, H. Q. , Y. Z. Zhang , Y. Y. Song , W. L. Tan , and Z. T. Qiu . 2017 Marital status is an independent prognostic factor for pancreatic neuroendocrine tumors patients: an analysis of the surveillance, epidemiology, and end results (SEER) database. Clin. Res. Hepatol. Gastroenterol. 41:476–486. 28416359 10.1016/j.clinre.2017.02.008

[15] Li, J. , B. E. Hansen , M. P. Peppelenbosch , R. A. De Man , Q. W. Pan , and D. Sprengers . 2017 Factors associated with ethnical disparity in overall survival for patients with hepatocellular carcinoma. Oncotarget 8:15193–15204. 28122352 10.18632/oncotarget.14771 PMC5362478

[16] Bilimoria, K. Y. , A. K. Stewart , D. P. Winchester , and C. Y. Ko . 2008 The National Cancer Data Base: a powerful initiative to improve cancer care in the United States. Ann. Surg. Oncol. 15:683–690. 18183467 10.1245/s10434-007-9747-3 PMC2234447

[17] Kloppel, G. , G. Rindi , A. Perren , P. Komminoth , and D. S. Klimstra . 2010 The ENETS and AJCC/UICC TNM classifications of the neuroendocrine tumors of the gastrointestinal tract and the pancreas: a statement. Virchows Arch. 456:595–597. 20422210 10.1007/s00428-010-0924-6

[18] Yao, J. C. , M. Hassan , A. Phan , C. Dagohoy , C. Leary , J. E. Mares , et al. 2008 One hundred years after “carcinoid”: epidemiology of and prognostic factors for neuroendocrine tumors in 35,825 cases in the United States. J. Clin. Oncol. 26:3063–3072. 18565894 10.1200/JCO.2007.15.4377

[19] Dasari, A. , C. Shen , D. Halperin , B. Zhao , S. Zhou , Y. Xu , T. Shih , et al. 2017 Trends in the incidence, prevalence, and survival outcomes in patients with neuroendocrine tumors in the United States. JAMA Oncol. https://doi.org/10.1001/jamaoncol.2017.0589 [Epub ahead of print]. 10.1001/jamaoncol.2017.0589 PMC5824320 28448665

[20] Rendall, M. S. , M. M. Weden , M. M. Favreault , and H. Waldron . 2011 The protective effect of marriage for survival: a review and update. Demography 48:481–506. 21526396 10.1007/s13524-011-0032-5

[21] DeSantis, C. E. , R. L. Siegel , A. G. Sauer , K. D. Miller , S. A. Fedewa , K. I. Alcaraz , et al. 2016 Cancer statistics for African Americans, 2016: progress and opportunities in reducing racial disparities. CA Cancer J. Clin. 66:290–308. 26910411 10.3322/caac.21340

[22] Schaefer, E. W. , M. Z. Wilson , D. Goldenberg , H. Mackley , W. Koch , and C. S. Hollenbeak . 2015 Effect of marriage on outcomes for elderly patients with head and neck cancer. Head Neck 37:735–742. 24596027 10.1002/hed.23657

[23] Wang, L. , S. E. Wilson , D. B. Stewart , and C. S. Hollenbeak . 2011 Marital status and colon cancer outcomes in US surveillance, epidemiology and end results registries: does marriage affect cancer survival by gender and stage? Cancer Epidemiol. 35:417–422. 21466984 10.1016/j.canep.2011.02.004

[24] Martinez, M. E. , K. Anderson , J. D. Murphy , S. Hurley S , A. J. Canchola , T. H. Keegan , et al. 2016 Differences in marital status and mortality by race/ethnicity and nativity among California cancer patients. Cancer 122:1570–1578. 27065455 10.1002/cncr.29886 PMC5523959

[25] Gomez, S. L. , S. Hurley , A. J. Canchola , T. H. Keegan , I. Cheng , J. D. Murphy , et al. 2016 Effects of marital status and economic resources on survival after cancer: a population‐based study. Cancer 122:1618–1625. 27065317 10.1002/cncr.29885 PMC5558592

[26] Wang, X. D. , J. J. Qian , D. S. Bai , Z. N. Li , G. Q. Jiang , and J. Yao . 2016 Marital status independently predicts pancreatic cancer survival in patients treated with surgical resection: an analysis of the SEER database. Oncotarget 7:24880–24887. 27036036 10.18632/oncotarget.8467 PMC5029750

[27] Jamii St. Julien, J. H. , and T. Ya‐Chen 2016 Impact of race on surgical management of pancreatic neuroendocrine tumors. J. Clin. Oncol. 34(Suppl 15):e18081.

[28] Liu, J. H. , D. S. Zingmond , M. L. McGory , N. F. SooHoo , S. L. Ettner , R. H. Brook , et al. 2006 Disparities in the utilization of high‐volume hospitals for complex surgery. JAMA 296:1973–1980. 17062860 10.1001/jama.296.16.1973

[29] Williams, D. R. , S. A. Mohammed , J. Leavell , and C. Collins . 2010 Race, socioeconomic status, and health: complexities, ongoing challenges, and research opportunities. Ann. N. Y. Acad. Sci. 1186:69–101. 20201869 10.1111/j.1749-6632.2009.05339.x PMC3442603

[30] Hashimoto, Y. , M. Shiina , T. Kato , S. Yamamura , Y. Tanaka , S. Majid , et al. 2017 The role of miR‐24 as a race related genetic factor in prostate cancer. Oncotarget 8:16581–16593. 28157714 10.18632/oncotarget.15016 PMC5369986

[31] Thompson, I. , C. Tangen , A. Tolcher , E. Crawford , M. Eisenberger , and C. Moinpour . 2001 Association of African–American ethnic background with survival in men with metastatic prostate cancer. J. Natl Cancer Inst. 93:219–225. 11158191 10.1093/jnci/93.3.219

[32] D'Arcy, M. , J. Fleming , W. R. Robinson , E. L. Kirk , C. M. Perou , and M. A. Troester . 2015 Race‐associated biological differences among Luminal A breast tumors. Breast Cancer Res. Treat. 152:437–448. 26109344 10.1007/s10549-015-3474-4 PMC4527078

[33] Manojlovic, Z. Comprehensive molecular studies of multiple myeloma in African Americans. 2016 AACR Conference on the Science of Cancer Health Disparities in Racial/Ethnic Minorities and the Medically Underserved. Presented −September 26, 2016.

[34] Krishnan, B. , T. L. Rose , J. Kardos , M. I. Milowsky , and W. Y. Kim . 2016 Intrinsic genomic differences between African American and white patients with clear cell renal cell carcinoma. JAMA Oncol. 2:664–667. 10.1001/jamaoncol.2016.0005 PMC5035632 27010573

[35] Ramakodi, M. P. , K. Devarajan , E. Blackman , D. Gibbs , D. Luce D , J. Deloumeaux , et al. 2017 Integrative genomic analysis identifies ancestry‐related expression quantitative trait loci on DNA polymerase beta and supports the association of genetic ancestry with survival disparities in head and neck squamous cell carcinoma. Cancer 123:849–860. 27906459 10.1002/cncr.30457 PMC5319896

